# Clinical consequences of human evolution shaped by cultural trends

**DOI:** 10.1093/emph/eos006

**Published:** 2013-01-03

**Authors:** Neil S. Greenspan

**Affiliations:** Department of Pathology, Case Western Reserve University School of Medicine, Cleveland, OH 44106, USA.

**Keywords:** human evolution, culture, genetic variation, selection, disease

## Abstract

Recent reports suggest that increased human population size, decreased negative selection pertaining to some phenotypes and associated genotypes and a possibly increased *de novo* mutation burden for newborns that relates to paternal age at conception are contributing to an expansion of human genetic diversity. Some of this diversity can be expected to contribute to disease. Because all of the preceding diversity-enhancing factors are to a significant degree consequences of cultural developments, it can be argued that the future clinical burden of the human population will be shaped in part by a human evolutionary trajectory substantially influenced by culturally mediated effects on the number of mutations in the gene pool and on the intensity of selection on some of the phenotypes associated with new genetic variants.

Several recent studies have added, or at least highlighted, a twist to the rather familiar conception of human evolution and this new variation on the standard theme may have substantial implications for both biomedical research and clinical medicine. Two of these studies [1, 2], published this past July, determined the nucleotide sequences of thousands or hundreds of human protein-coding genes in thousands of people. What their data revealed was that there were many rare (frequency <0.5%) genetic variants, most of which were previously unknown and relatively localized geographically or ethnically. These mutations were enriched among individuals with diagnosable medical conditions.

Although there may always be some holdouts, especially among non-experts and non-scientists, most biomedical scientists and biologists recognize that humans have not stopped evolving and continue to be subject to selection and to change at a population level, however slowly or subtly. In terms of both genotypes [3–5] and phenotypes [6], there is growing evidence for human evolution in response to selection.

The authors of both of the recent studies on the prevalence of rare genetic variants cited above inferred that many of the rare variants they described were surviving in the human population in part because selection against certain types of functional deficits was diminished in recent decades in comparison with the past. Examples of medical advances that have saved many lives and likely permitted the generation of offspring who would not otherwise have been born include blood and marrow transplantation for pediatric lymphoid malignancies and vaccines against pediatric pathogens capable of causing mortality. Since 1971, over a million individuals have received hematopoietic cell transplants, generally for otherwise fatal diseases [7]. A significant proportion of these recipients were of reproductive age or younger. The implementation of routine immunization for diphtheria, mumps, pertussis and tetanus resulted in a greater than 99% reduction in mortality from these infectious diseases between 1940 and 2004 [8]. If instead we consider a disease directly associated with mutations at a single locus, such as cystic fibrosis (CF), the improvement in mortality over the past 40 years is also highly significant. For example, in the UK, in the period from 1968 to 1970, approximately half of the population of males or females succumbed to the disease by the time of entry into reproductive competency, but by the early 1990s, the majority of UK CF patients could be expected to survive well into their reproductive years [9]. Similar data have been obtained in Australian CF patients [10]. There are undoubtedly a number of other potentially fatal conditions associated with one or more alleles at a single predominant genetic locus where improvements in care have increased survival and reproductive success.

In part as a consequence of the contributions of public health measures and advances in medical care, an increased pace of population growth has been sustained for many generations and has made available many new genetic variants for which selection has had insufficient time to act irrespective of any attenuation of selection intensity. The enormously expanded human population of recent decades has also meant that there are many more opportunities for genomes to sample what might be regarded as the envelope of human genetic possibility [11]. It is sobering to realize that, starting with any particular human genome, the number of possible genomes one mutational step away is three raised to roughly the three billionth power, a number staggeringly larger than estimates for the number of atoms in the universe (which generally cluster around 10^79^ to 10^80^), a reasonable gold standard for impressive magnitudes [12].

Another even more recent study [13] found that the number of new mutations in offspring is strongly correlated with the age of the father at the time of conception. This correlation helps to explain the substantial correlation between the occurrence of *de novo* mutation and the incidence of autism [14–16]. According to Kong *et al.*, the average newborn has 60 new small-scale mutations, but the paternal contribution can vary over a wide range from about 25 for a 20-year-old father to 65 for a 40-year-old father with a fairly constant 15 new mutations contributed by the mother. Available evidence suggests that up to 10% of new point mutations are expected to be deleterious [17], so it is expected that, on average, each newborn could have as many as six new potentially disease-causing genetic alterations.

One far-reaching implication of these new results is that personalized medicine pertaining to some conditions will likely face greater obstacles than previously believed. Establishing the causal connections between rare variants found in geographically circumscribed populations and diseases or other medically relevant phenotypes will be much more difficult, requiring much larger study sample sizes for example, than has been the case for more common variants that occur in multiple populations on different continents. Furthermore, once such causal links are established, developing relevant genetically guided diagnostic tests or therapies could be more challenging than has generally been assumed.

Thus, the crux of the current thesis is (i) due to cultural developments such as technologically advanced medical care, public health measures and increased food availability, there are many more human genomes subject to mutation than there otherwise would be and (ii) many new variants that in a context with less medical technology, public health infrastructure and food availability would be much more likely to disappear quickly now persist in the human population. So cultural developments have made it possible for more human genetic variants to arise and, by relaxing selection on many of the variant-associated phenotypes, for more of these variants to persist in the human genome pool. A culturally mediated increase in mutation rate may further enhance this process but is not essential for its general direction.

Of course, strong interactions between cultural evolution and biological evolution have been noted before, and the empirical evidence supporting such interactions is consistent with theoretical analyses [18]. A now widely cited and well-accepted example of cultural influence on human evolution is the effect of dairying on the frequency of alleles that favor the adult persistence of lactase expression in the intestines [19]. In this study, Tishkoff *et al.* reported evidence implicating several independently originating variants associated with lactase persistence in European and African populations due to positive selection.

The new data, in contrast, reveal the persistence and possible spread of variants primarily due to a lack of selection resulting from cultural factors. These results further support the argument that cultural and biological forms of evolution are better regarded as a single integrated process than as separate influences on human populations [18, 20]. Physicians who understand these realities will be better able to understand the eternally changing spectrum of human disease and disability.
